# Adaptive modes of adult men during COVID-19: qualitative analysis under Roy's model*

**DOI:** 10.17533/udea.iee.v40n3e14

**Published:** 2023-02-14

**Authors:** Vinícius de Oliveira Muniz, Anderson Reis de Sousa, Thiago da Silva Santana, Alisson dos Anjos Santos, Ramon Evangelista Luz, Eric Santos Almeida, Isabella Félix Meira Araújo, Evanilda Souza de Santana Carvalho

**Affiliations:** 1 Nurse, Master. Ensinar Brasil Institute. Serra, ES, Brazil. Email: vinciusomuniz22@gmail.com. Corresponding author. Ensinar Brasil Institute Serra ES Brazil vinciusomuniz22@gmail.com.; 2 Nurse, PhD. Federal University of Bahia (UFBA). Salvador Bahia Brazil Email: anderson.sousa@ufba.br Universidade Federal da Bahia Federal University of Bahia Salvador Bahia Brazil anderson.sousa@ufba.br; 3 Nurse, Master. State University of Feira de Santana (UEFS). Feira de Santana, Bahia, Brazil Email: tssantana@uefs.br Universidade Estadual de Feira de Santana State University of Feira de Santana Feira de Santana Bahia Brazil tssantana@uefs.br; 4 Medical student, Bachelor of Health. Federal University of Bahia (UFBA). Salvador Bahia Brazil Email: alissons@ufba.br Universidade Federal da Bahia Federal University of Bahia Salvador Bahia Brazil alissons@ufba.br; 5 Nurse, Specialist. United Faculties of Research, Science and Health (FAPEC), School of Nursing. Salvador Bahia Brazil. Email: ramonlluz@hotmail.com United Faculties of Research, Science and Health School of Nursing Salvador Bahia Brazil ramonlluz@hotmail.com; 6 Nurse, Master. Federal University of Bahia (UFBA). Salvador Bahia Brazil Email: eriksdn@gmail.com Universidade Federal da Bahia Federal University of Bahia Salvador Bahia Brazil eriksdn@gmail.com; 7 Nurse, Master. Federal University of Bahia (UFBA). Salvador Bahia Brazil Email: isabellafelixmeira@hotmail.com Universidade Federal da Bahia Federal University of Bahia Salvador Bahia Brazil isabellafelixmeira@hotmail.com; 8 Nurse, PhD. State University of Feira de Santana (UEFS). Feira de Santana, Bahia, Brazil Email: evasscarvalho@uefs.br Universidade Estadual de Feira de Santana State University of Feira de Santana Feira de Santana Bahia Brazil evasscarvalho@uefs.br

**Keywords:** pandemics, COVID-19, adaptation, men's health, nursing theory., pandemias, COVID-19, adaptación, salud del hombre, teoría de la enfermería., pandemias, COVID-19, adaptação, saúde do homem, teoria de enfermagem.

## Abstract

**Objective.:**

This study aims to understand how adult men adapt to the COVID-19 pandemic.

**Methods.:**

Qualitative study involving 45 adult men residing in Brazil in 2020. Data were obtained from a Web Survey and treated using Reflective Thematic Analysis and interpreted in the light of Callista Roy's Adaptation Model.

**Results.:**

The COVID-19 pandemic mobilized in men the ways of adaptation that are configured in: mobilization of the physiological-physical and regulatory dimension: adjustments in the sleep pattern, dietary pattern, and maintenance of physical activity; group self-concept identity: managing emotions; role function: self-knowledge and self-care; interdependence: adjustments in the marital relationship, family ties and paternity, investment in training and studies and control of excessive consumption of content on cell phones.

**Conclusion.:**

The perception of the own vulnerability favored the entry of men into ways of adaptation in search of balance during the pandemic, motivating them to move through practices of taking care of themselves and taking care of others. Markers of psycho-emotional distress alert to adherence to new modes of care capable of promoting healthy transitions in the face of disruptions and uncertainties generated by the pandemic. This evidence can support the establishment of goals for nursing care aimed at men.

## Resumen

**Objetivo.:**

Comprender cómo se estructuran los modos de adaptación de los hombres adultos a la pandemia de COVID-19.

**Métodos.:**

Estudio cualitativo que involucró 45 hombres adultos en Brasil en 2020. Los datos se recolectaron mediante la Encuesta Web y a continuación se sometieron al Análisis Temático Reflexivo e interpretados bajo el Modelo de Adaptación de Callista Roy.

**Resultados.:**

La pandemia de COVID-19 movilizó en los hombres modos de adaptación que se configuran en: movilización de la dimensión fisiológico-física y reguladora: ajustes en el patrón del sueño, la alimentación y el mantenimiento de la actividad física; autoconcepto grupal de identidad: gestión de las emociones; función de rol: autoconocimiento y autocuidado; interdependencia: ajustes en la relación conyugal, vinculación familiar y paternidad, inversión en formación y estudios, y control del consumo excesivo de contenidos en el dispositivo móvil.

**Conclusión.:**

La percepción de la propia vulnerabilidad favoreció el ingreso de los hombres a modos de adaptación en busca del equilibrio durante la pandemia, lo que los estimuló a transitar por prácticas de cuidarse a sí mismos y cuidar de los demás. Los marcadores de sufrimiento psicoemocional alertan sobre la adhesión a nuevos modos de atención capaces de promover transiciones saludables ante las disrupciones e incertidumbres generadas por la pandemia. Esta evidencia puede apoyar el establecimiento de metas para el cuidado de enfermería hacia los hombres.

## Resumo

**Objetivo.:**

Compreender como se estruturam os modos de adaptação de homens adultos à pandemia da COVID-19.

**Métodos.:**

Estudo qualitativo que envolveu 45 homens adultos residentes no Brasil em 2020. Os dados foram obtidos de W*eb Survey* e tratados mediante Análise Temática Reflexiva e interpretados à luz do Modelo de Adaptação de Callista Roy.

**Resultados.:**

A pandemia da COVID-19 mobilizou nos homens os modos de adaptação que se configuram em: mobilização da dimensão fisiológico-física e regulatória: ajustes no padrão de sono, alimentar e manutenção da atividade física; identidade do autoconceito de grupo: gestão das emoções; função de papel: autoconhecimento e autocuidado; interdependência: ajustes no relacionamento conjugal, vinculação familiar e na paternidade, investimento na formação e estudos e controle do consumo excessivo de conteúdos no aparelho celular.

**Conclusão.:**

A percepção da própria vulnerabilidade favoreceu a entrada dos homens em modos de adaptação em busca de equilíbrio durante a pandemia, motivando-os a transitar por práticas de cuidar de si e cuidar do outro. Marcadores de sofrimento psicoemocional alertam para adesão a novos modos de cuidado capazes de promover transições saudáveis diante das rupturas e incertezas geradas pela pandemia. Essas evidencias podem apoiar o estabelecimento de metas para o cuidado de Enfermagem dirigido aos homens.

## Introduction

On a large scale, men have experienced an unprecedented experience that transformed the universe as a result of the impacts caused by COVID-19, declared a pandemic on March 11, 2020.(1) Faced with the scenario of uncertainties, significant social and environmental disruptions, the subjects were mobilized to promote new physiological and psychosocial adaptive modes.(2) Because it is a highly transmissible disease, health authorities established collective social distancing as a priority measure for the containment of the virus, however, this measure precipitated the rupture of socio-affective networks, financial balance and changes in human productive capacity, which abruptly began to experience anticipatory grief. As a result, routines were redesigned, others established and the various commitments, especially labor, began to be developed remotely on a large scale.(3)

Until November 2021, in Brazil, there were nineteen million people with confirmed diagnoses and a total of 608,671 deaths resulting from COVID-19,(4) a study carried out in a Brazilian state showed that the case fatality rate was only among men 2.71%, while among women, 1.48%.(5) This same scenario is observed in other countries and has challenged researchers and policymakers to understand the reasons why the cisgender male audience has been more affected.(6)

Despite evidence that showed different modulations of the immune response between men and women,(7,8) understanding the higher rates of infections among men presupposes the social dimension in which the experience of illness reflects the intersections of other elements, which impact on health behaviors causing greater male vulnerability to contamination by COVID-19, which can be modified requiring goals for care.(9,10)

In addition, the fact that the complications of COVID-19 have repercussions on the bioenergetic dysfunction of men, evident in post-Covid-19 disabling syndromes,(6,7) aroused our interest in proposing adaptive care of nurses, for which we chose as a theoretical reference the Roy's Adaptation Model (RAM),(2) as it enables the achievement of interventional specificities in the production of care for men, according to its adaptive modes, which interact with the adaptive regulatory and cognition subsystems to face the improbabilities experienced by men during the pandemic.(2,10-12) Thus, this article was guided by the following research question: How has the COVID-19 pandemic produced/mobilized the emergence of adaptive modes among adult men? Its objective was: to understand the ways in which adult men adapt to the COVID-19 pandemic, based on the model proposed by Callista Roy.

## Methods

This is a qualitative research structured in the light of the theoretical framework of Roy's Adaptation Model (RAM), which aims to maintain a preserved state of health amidst the changes that have taken place, with full appreciation of people who face new imposed challenges such as the health crisis(2,13) This study has a multicenter character and is part of a matrix project of national scope, for which the virtual environment (Web Survey) was used to develop data collection, through a semi-structured and self-administered form, available on the Google Forms^®^ platform, whose answer took about 20 minutes and contained closed and open questions related to sociodemographic, work, health conditions and the phenomenon of interest to the investigation, in the form of the following questions: How have you been experiencing the COVID-19 pandemic? What have you been doing to face the COVID-19 pandemic?

The data collection instrument was submitted to internal validation (pilot test) by two PhD researchers, a special PhD student and two regular PhD graduate students, and externally by a group of 25 participants (seeds) chosen convenience for being available to participate in the research in all its phases. Data were collected between June and September 2020 with a sample of 45 men over 18 years of age, who met the inclusion criteria: consider themselves men, be an adult and reside in Brazil in the context of the pandemic; those participants who found on international trips during this period were excluded.

The research team was composed of four researchers (men) and two researchers (women), with experience in the research area, working in teaching, research and service during data collection, without contact and/or direct link with the participants who maintained all technical rigor and scientific required in qualitative research, obtaining the study design according to the COREQ recommendations. To identify possible participants, the strategy of consecutive recruitment of men was adopted using the Snowball technique,(14) in which a group of the first 25 participants who responded to the invitation, called “seeds”, were accessed and later shared the hyperlink for other men.

The approach and recruitment took place through social networks such as Facebook®, Instagram®, WhatsApp®, Grindr® and Scruff®. Dating apps were selected because they had a high concentration of male users, so informal messages were sent asking if they were interested in answering the form, and when the answer was productive, the hyperlink was sent. In the social media of the project, inviting layouts related to the research were disclosed.

The process of theoretical exhaustion(15,16) took place from the interruption of data collection because they were no longer inferred and because they did not bring new clarifications to the object of study investigated in the observed field. To preserve the anonymity of the participants, they were identified by the letter M and followed by randomly distributed Arabic numerals.

To assist in the systematization of the data, the NVIVO12 Software was used. Data analysis followed the deductive perspective, guided by the theoretical/methodological proposal contemplated in Roy's four adaptive modes: 1) physiological-physical (physical and chemical processes of human activities in the face of adaptations); 2) group self-concept identity (psycho-spiritual integrity with the sense of being unity and its purpose in the universe); 3) role function (roles held in society and social integrity) and 4) interdependence (structural relationship of the solitary or collective being);(2,13) as well as by the Reflective Thematic Content Analysis proposed by Clark and Braun, in its six stages: rigorous reading and re-reading of data; creation of theoretical codes (nodes); derivation of emerging themes and subthemes; code grouping; search for relationships between the themes that emerged; naming of themes and development of the analysis synthesis corresponding to the object of investigation.(17)

The project was approved by the Research Ethics Committee of the Federal University of Bahia, under opinion number: 4,087,611 and CAAE: 32889420.9.0000.5531, according to resolutions 466/12 and 674/2022 and Circular Letter 2/2021, of the CNS, in addition to the general data protection law. The participants declared their consent to the Informed Consent Term (ICT) in the imagery modality. For the preservation of identity, codes were created for the participants, such as: M1 (Man 1), M2 to M45.

## Results

Most of the individuals involved in this study identified themselves as heterosexual, cisgender, of mixed race/color, with a higher education level, with a mean salary income above five minimum wages, single, living in urban areas, living with family members, not elderly. Participants reported not having been affected by COVID-19 or having sequelae that could be attributed to the disease. They used both the Unified Health System and the private subsystem to access general health services and care. The empirical material was framed in four Thematic Analysis Categories (TAC) in convergence with RAM, namely: Physiological-physical mode and regulatory subsystem (TAC1), composed of 3 Thematic Cores (TC): changes in sleep pattern (TC1A), changes in dietary pattern (TC1B), and changes and adoption of measures to maintain physical activity (TC1C); Group self-concept identity mode (TAC2), with 01 TC: changes in the management of emotions (TC2A); Role function mode (TAC3), with 01 TC: improvement of self-knowledge, self-care and health care practices (TC3A); Change of interdependence (TAC4) with 4 TC: Changes in the dynamics of the marital relationship, in the performance of roles and in the exercise of paternity (TC4A), Improved strengthening of family ties (TC4B), Increased availability of time to study (TC4C), and excessive cell phone use (TC4D).

### TAC1 - Physiological-physical mode and regulatory subsystem.

Dimensions such as dietary patterns, physical activity and sleep constantly fluctuated among men, both for the decline and for the achievement of favorable advances in these patterns. The pandemic context gave rise to the physiological-physical mode, which manifested the construction of physical and organic processes closely linked to body systems and the adoption of operational resources considered basic, also understood as regulatory coping subsystems. 

### TC1A: Changes in sleep pattern

*I am changing night for day. My sleep is totally altered. Much has changed since the beginning of the pandemic*. *Because of this I've been trying to sleep early.* M1; 

*there have been changes in my sleep. I have been having trouble sleeping and am waking up earlier than usual. Therefore, I am avoiding consuming news about Covid-19 and using my cell phone at night.* M4; 

*I am changing night for day.* M15; 

*I have been having insomnia and this has led me to adopt sleep hygiene*. M22. 

### TC1B: Changes in dietary pattern

*there are days when I have been eating a lot and days when I have absolutely no appetite. This has changed my weight, because sometimes I gain weight, sometimes I lose weight suddenly. Because of this, I started to weigh myself indoors and control the consumption of unhealthy foods.* M2; 

*there are days that I eat a lot of what I ate before the pandemic, mainly because of anxiety. To avoid overconsumption of food I have been trying to control anxiety*. M16; 

*the stress of the pandemic made me eat more than usual and I had to seek guidance from a nutritionist in a virtual way*. M21; 

*I started to take care of my diet to improve physical health I started to consume vitamin supplements, foods rich in vitamins and sunbathing*. M44. 

### TC1C: Adoption of measures to maintain physical activity

*because I spend more time indoors, I have been doing weight training at my house since the pandemic started, because I'm afraid of losing my physical shape, which is super important to me*. M22; 

 I *was very sedentary, stressed, and because of that, I went back to physical activity, even indoors, as an escape valve to deal with this change in routine*. M23; 

*I'm more nervous, I stopped doing physical activities at the gym, but I tried to adapt as best I could, doing exercises at home, watching videos on the internet*. M34. 

### TAC2 - Group self-concept identity mode

 Disorders of a socio-psychological nature were expressed through reports of anxiety, panic attacks, stress, irritability, mood swings, excessive worry, tension and distress. The identity of the group self-concept refers to some components such as psychological and spiritual integrity and the sense of unity with a purpose in the universe that are combined. 

### TC2A: Changes in managing emotions

*there have been several changes since the beginning of the pandemic. I started to have emotional changes such as the appearance of anxiety and panic attacks, which made me understand myself better and also the situations around me. I have been more aware of my emotional state than before*. M6; 

*I am less reactive and more reflective, trying daily to control stress and keep myself calm*. M12; 

 the isolation made me more anxious and before that I have taken care of my mental health to control anxiety. M28; 

*the accumulation of tense days has contributed to my bursting point. After the pandemic, I started to pay more attention to my mental health and control my behavior.*M38;

*with isolation and reclusion I've been living a physical and mental mess and I've been trying to organize my mind*. M39; 

*I noticed some changes in my mood. I have been feeling very tense, worried, distressed with the current situation, especially in the work environment. In view of this, I have sought to share problems with friends, have moments of leisure and be closer to my family*. M43;

*in order to protect my mental health I started to meditate, read and have more interaction with those closest to me*. M45. 

### TAC3 - Role function mode

Obtaining greater personal care was reported along with the acceptance process that has been taking place in the daily lives of men who have come to better understand the news reported. The data brought from the roles occupied by the participants in society and the reflection on “who I am before others”, gave rise to a category and a thematic nucleus. 

### TC3A: Improvements in self-knowledge, self-care and health care practices

*the pandemic changed everything about me. I started to occupy my mind with activities that I didn't do before, and I feel more useful, creative and supportive.* M27; 

*it has been a moment of greater understanding of the "I" within. More patience with the other members where I live. Be more communicative despite isolation. Greater confidence in the "I" figure, perception of my ability as a person, human being, professional*. M33; 

*my personality was completely transformed. Today I have experienced greater empathy, a greater sense of need for giving and collaboration (which I have done in many ways), greater tolerance and compassion, and an even better relationship with my wife*. M35; 

*I am understanding better when being open to conversation is important to resolve possible impasses and I have learned to deal with my bad moments*. M36; 

*I believe that I am living more intensely everything that I have.* M37; 

*I have learned to value the little things more and pay attention to what I didn't give so much importance before*. M41. 

*I'm taking more care of myself and others.* M8; 

*after Covid-19 I started to improve prevention care*. M9; 

*I'm more careful about hygiene. I started washing my hands more and greeting people from afar while maintaining social distance.* M14; 

*I have taken care of the new routine and management of my days, optimizing my time at home better*. M26; 

*I learned to seek, within myself, comfort to deal with my anguish. In accepting what is going on inside of me, I was very likely to deny anything that was going on inside of me. I learned to look for resources, activities that bring more integration such as yoga and to have healthier habits in my daily life. Not to mention respecting other people's spaces, having more patience and sincerity with my feelings*. M32; 

*I have been trying to get to know myself more and better. I noticed that I started to be more patient and less blown away. I learned to connect more with people virtually and listen to each one of them with real interest and willingness.* M42. 

### TAC4 - Interdependence Mode

There were reports of greater marital loneliness due to lack of communication and the perception of new experiences such as sharing household chores with partners and longer caring for children. Social isolation was consciously and co-responsibly assumed, and the men participating in this study maintained their affective concerns and perceived greater approximation, interaction and communication with their families. New discoveries on how to study and work in the same environment were cited positively, but demotivation and changes in mental health emerged as consequences of this situation. Some started reading, studied more and used their cell phone excessively without time control. 

The four cognitive-emotive channels: perceptual and information processing, learning, judgment and emotion were identified in the data due to dynamic processes that synthesized specific axes. 

### TC4A: Changes in the dynamics of the marital relationship, in the performance of roles and in the exercise of paternity

*I feel that lately I've been more isolated from my partner, it's just the two of us at home, and there are days that we stay all day without speaking properly. In view of this, I started to strengthen affective bonds, seeking to be closer, available to support her and to exchange affection in this difficult time that the pandemic is being*. M7; 

*I have experienced many changes in the routine in relation to household chores, in addition to caring for children during the period of social isolation. I sought to talk to other men who are married and who are also fathers to find out how they were dealing with this situation. I have tried to participate in the care of the children and the house, making the family more united.* M40. 

### TC4B: Improved strengthening of family ties

*the pandemic brought a great distance from my family, but I have been more concerned about my family and closest people. As I am exposed to the virus, I need to take care that was not necessary before (restriction of contact with father and mother mainly. In addition, I always try to ask those closest to me how they are, how they feel. I exchange experiences with the family, to facilitate this whole moment*. M11; 

*I'm closer to my family and with that I talk more with them.* M24; 

*in the beginning, I felt more productive and motivated to work, today I feel a little more unmotivated, but I have been trying to cope with the responsibilities I took on during this period.* M30; 

*in addition to working from home, the changes have positive practical points, but also negative ones, such as affecting my mental health.* M31. 

### TC4C: Investment in training and optimization of time to study

*the pandemic brought many difficulties in my study and work routine. As a result, I sought to adhere to new ways of studying and working, avoiding changes in my ability to work. at home, I looked for new ways to learn, approaching the new modalities of virtual teaching.* M13; 

*I tried to prioritize my studies, as I have had more free time at home to study*. M17; 

*because of the isolation I tried to read and attend virtual classes, much more than before*. M18; 

*I dedicated myself to reading more, however as a way of escaping from reality.* M19; 

*I started to study more about public policies aimed at health.* M20

### TC4D: Control over cell phone usage

*throughout the day I have spent more time on my cell phone than necessary. The use has become excessive and I'm having trouble reducing the length of stay. Because of that I started to decrease the usage time, I removed the notifications and sound messages*. M3. 

*with the pandemic I started to feel changes in my individual organization in relation to daily activities. Now I have been trying to control the consumption of content on my cell phone, I avoid using it at night and reducing the frequency of access on social networks*. M5. 

**Figure 1:**
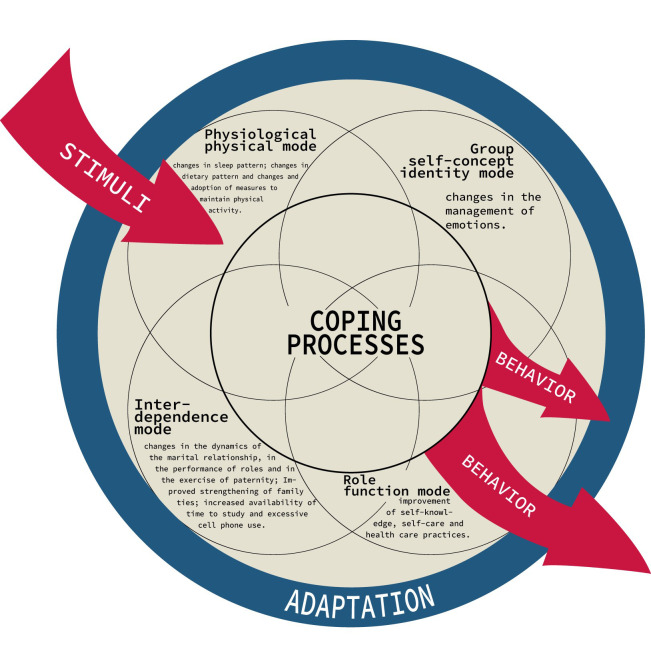
Diagrammatic representation of human adaptive systems to the experience of adult men in the Covid-19 pandemic in Brazil, Salvador, BA, Brazil, 2020.

Source: Adapted from Russian; Baumann; Velasco-Whetsell; Roy, 2020.(2)

The experience of men in the COVID-19 pandemic mobilized the construction of adaptive modes according to RAM adapted based on the findings found, which can be seen in Figure 1, which represents a diagram of human adaptive systems in which there is a centralization of the coping process surrounded by four modes of adaptation that generated new behaviors.

## Discussion

The analysis of male adaptation in the face of the COVID-19 pandemic made it possible to perceive that their ways were convergent, which made it possible to carry out the theoretical framework of RAM; however, they reserve symbolic spaces of the uniqueness of each subject in the face of the experience of the "new self" of the pandemic, which could be observed in the oscillations of health patterns in the physiological-physical mode and in the adaptation regulatory subsystem. New findings are evidenced, such as the openness of men to concern for a healthy adaptation and transition in the face of the numerous changes in their respective social and family contexts through attitudes that provide a healthier life in the face of the pandemic. Mental health stands out, stressing for self-care, in mobilizing adaptive modes and relating to the daily dynamism in which men reported accessing reliable sources of news about the pandemic and filtering the media excess of social networks; they also started integrative practices (meditation and physical activity) and therapeutic activities in male groups in a virtual environment to circumvent the psychosocial impacts.(18) This means that men express alternatives in the face of the perception of their own vulnerability and the feelings of fragility that enhance the effective search for caring for themselves and for others. (6,11,18)

The cognitive-emotive and regulatory channels of RAM(2,13) in view of the findings of this study, were manifested from the perceptive processing of information, learning, judgment and emotion of the adaptive process issues that respond to the dynamics of facts through the neural, chemical and endocrine coping channels. This can be seen in the expressions of self-perception regarding the impacts suffered by men.(18) Although some men have reported alternative measures of low adherence among the male population to deal with the process of coping and adaptation, such as meditation and yoga, others have highlighted the weaknesses in the sleep pattern, with emphasis on insomnia and changes in appetite, which included reduced and increased desire to eat. Thus, men with a higher level of education, economic and work and with greater ease of access to private health services may have a favorable adaptation to achieve balance even in the pandemic.(18) Therefore, the link between sociodemographic conditions and effective adaptive forms it becomes more understandable.(19)

In general, alterations in sleep patterns, in the irregularity between sleeping and waking up, are risk factors for mental health disorders in all human beings, regardless of gender, which reflect a decline in the capacity for intellectual and cognitive function performance.(20) The relationship between men reporting problems in the sleep-wake cycle goes beyond the neurochemical mechanism, as the difficulty in sleeping, waking up and maintaining a desirable quality of sleep, establishes an interface with the excessive feeling of worry that is favorable for insomnia(20) due to confinement and the home office, which promoted the adaptive/regulatory mode by awakening a stimulus to its execution to preserve some immunological/emotional activities in men.(21)

However, an intersection between altered sleep patterns, mood/behavior changes and acute mental disorders, such as the tension/stress/nervousness triad, panic crisis, demotivation and depression, can already be observed. Depression, in turn, is bidirectionally associated with insomnia and makes its treatment a complex clinical management item. It is noteworthy that insomnia causes more depression and both must be treated concomitantly.(20) This situation alerts to the needs triggered by the restrictions and disruptions in the socialization of men that can, in addition to favoring mental illness, make it difficult to access and adhere to psychosocial support programs.

In pandemic contexts, the problem can worsen and make the adaptation process even more complex, which will require nursing interventions anchored in the philosophical and cultural assumptions of RAM,(2,13) aimed at guidance on sleep hygiene, promotion of healthy eating with reduced consumption of alcohol, other drugs and caffeine, and greater control over access to electronic objects before going to bed, as excessive cell phone use without control over time was evidenced.(20,21)

Changes in dietary patterns also emerged in this period and had negative impacts on men's health and quality of life. Such changes are related to mental health aspects and have repercussions on a dysfunctional diet in the face of the psycho-emotional responses mobilized in the pandemic, since the problem of sedentary lifestyle, permeated by physical inactivity, increased time in the sitting position and excessive food intake, raising the possibility that men developed morbidities from the group of Chronic Non-communicable Diseases (CNCDs).(22)

Taking into account the behaviors and attitudes of men's practices, adaptive modes have shown to be ascending, and need to be better explored by multidisciplinary teams, in particular the nurses, with a view to achieving tangible goals of care with other groups of men, namely: acquisition of healthy eating habits; prevention of health problems; hygiene; meditative practices; behavioral management of emotions; strengthening of affective bonds; virtual interaction/communication; hope; self-efficacy; self-compassion and establishment of socio-affective networks.

In addition, the “mode of interdependence”, which involves the structure and development of the person as a unit or his collectivity, in a dimension of the cognitive-emotional coping subsystem,(2,13) was present in the experience of men, and may be related to the way they processed the individual adaptability that caused marital, family and paternity changes, and that for these reasons, they lack nursing interventions to support the adaptation of affective relationships between couples and different partnerships, aiming to promote a good relationship, non-violence and a culture of peace with a focus on family harmony and balance.(12,19) It is worth mentioning that with the advance of vaccination, the population acquired subsidies to face an installed international disaster and made men return, as far as possible, with its socializations in different groups, with nursing as a promoter of safety under a collective gaze, in different formats, using technology as a vehicle for communication and digital social interaction.(23,24)

Regarding mid-range Nursing Theories, they are still poorly incorporated into care in the different complexities of health systems, which need to conform to the logic of complex adaptives, because when they are used, nursing theories create foundations that reformulate practices and protocols for the qualification of care, directing it to the specific demands of men during the pandemic and enabling the applicability of focal, contextual and residual stimuli belonging to Roy's model.(2,13)

Regarding the limitations of the study, there was the possibility of biases in relation to the sample in view of the recruitment technique used, the inaccessibility of groups of men with low levels of digital literacy or with limited access to technological resources such as a smartphone and the lack of deepening of knowledge given the impossibility of establishing a dialogue with the participants. Such facts, according to RAM, can hamper the process of monitoring what is adaptable or not, and prevents generalization.

Finally, nurses can expand the scope of their actions, when contemplating the concept “nursing goal”(2) by mobilizing the promotion of adaptation of the four adaptive modes proposed by Callista Roy that named the analytical categories of this study, and for carrying out investigations at the regulatory and cognition levels, as they define the problem more accurately and support the decision on which care plan will achieve its expected results.

## Conclusion

Faced with the emergence of the COVID-19 pandemic and its different consequences, men expressed ways of adapting to social interaction facilitated by digital connectivity, generating success in the cognitive subsystem. On the other hand, the group self-concept identity mode still remains in modulation and non-adaptation, especially in mental health, which has been altered, while physical health has been altered through a sedentary lifestyle, eating and sleeping patterns. The way of interdependence was manifested when exercising new behaviors, attitudes and practices that in general are directed to the management of emotions, self-knowledge, spirituality/transcendence and the strengthening of the construction of bonds through socio-affective networks. In addition, the need for adequacy and qualification of health care regarding the adaptation of men was identified, above all, the role that nursing has in providing analytical elements such as RAM, which allow the apprehension and subsidizing of interventions at the individual and collective levels that contribute to better assisting emerging demands.
